# 783 Consensus Criteria for Palliative Care Consults: A Modified Delphi Approach

**DOI:** 10.1093/jbcr/irad045.258

**Published:** 2023-08-29

**Authors:** Daniel H Grossoehme, Carrie Brown, Miraides Brown, Sarah Friebert, Anjay Khandelwal, Richard B Lou, Esther Teo

**Affiliations:** Akron Children’s Hospital, Akron, Ohio; University of Arkansas for Medical Sciences, Little Rock, Arkansas; Akron Children’s Hospital, Akron, Ohio; Akron Children’s Hospital, Akron, Ohio; Akron Children’s Hospital, Akron, Ohio; Akron Children’s Hospital, Akron, Ohio; University of Arkansas for Medical Sciences, Little Rock, Arkansas

## Abstract

**Introduction:**

Specialist palliative care (PC) is a limited resource, best conserved by identifying persons most likely to benefit from a PC consult. Little guidance is available for clinicians on whether/ when to consult PC. The authors previously developed empirically-derived consult criteria to guide burn providers in specialist PC consultation, refining them by expert panel input.

**Methods:**

A modified three-iteration Delphi process was employed to revise and develop consensus on empirically-derived PC consult criteria in burn patients. Eligible participants (N=202) were identified by (a) having published on burn and PC (b) presented nationally on burn and PC or (c) nomination by the study team as having equivalent expertise in the field.

**Results:**

Forty-three individuals participated in Iteration 1; n=34 (79%) in Iteration 3, which rank-ordered three versions of criteria and invited further revisions. Consensus eliminated the most parsimonious version. Expert review panelists integrated feedback, yielding a consensus list with 6 criteria for PC consultation and 6 criteria for consideration of PC consult. Consult criteria include patient/family request; conflicted or unclear goals of care; and high modified Baux/SCORTEN scores. Criteria for consideration include no longer meeting milestones; assistance with symptom management; or cardiac arrest.

**Conclusions:**

Consensus among burn and PC providers enhances validity and supports clinical use to guide burn providers on patients most likely to benefit from specialist PC consultation. Future research should prospectively explore whether adherence to the criteria is associated with improved outcomes and potential further revision.

**Applicability of Research to Practice:**

Provides guidance on PC consultation and may be used to examine outcomes.

